# Inhibition of human immunodeficiency virus type-1 by cdk inhibitors

**DOI:** 10.1186/1742-6405-7-7

**Published:** 2010-03-24

**Authors:** Irene Guendel, Emmanuel T Agbottah, Kylene Kehn-Hall, Fatah Kashanchi

**Affiliations:** 1 Department of Microbiology, Immunology, and Tropical Medicine, The George Washington University, Washington, DC, 20037, USA; 2 Department of Biochemistry and Molecular Biology, The George Washington University School of Medicine, Washington, DC, 20037, USA; 3 Department of Molecular and Microbiology, National Center for Biodefense & Infectious Diseases, George Mason University, Manassas, VA 20110, USA; 4 Keck Institute for Proteomics Technology and Applications, The George Washington University, Washington, DC, 20037, USA

## Abstract

Current therapy for human immunodeficiency virus (HIV-1) infection relies primarily on the administration of anti-retroviral nucleoside analogues, either alone or in combination with HIV-protease inhibitors. Although these drugs have a clinical benefit, continuous therapy with the drugs leads to drug-resistant strains of the virus. Recently, significant progress has been made towards the development of natural and synthetic agents that can directly inhibit HIV-1 replication or its essential enzymes. We previously reported on the pharmacological cyclin-dependent kinase inhibitor (PCI) *r*-roscovitine as a potential inhibitor of HIV-1 replication. PCIs are among the most promising novel antiviral agents to emerge over the past few years. Potent activity on viral replication combined with proliferation inhibition without the emergence of resistant viruses, which are normally observed in HAART patients; make PCIs ideal candidates for HIV-1 inhibition. To this end we evaluated twenty four cdk inhibitors for their effect on HIV-1 replication *in vitro*. Screening of these compounds identified alsterpaullone as the most potent inhibitor of HIV-1 with activity at 150 nM. We found that alsterpaullone effectively inhibits cdk2 activity in HIV-1 infected cells with a low IC_50 _compared to control uninfected cells. The effects of alsterpaullone were associated with suppression of cdk2 and cyclin expression. Combining both alsterpaullone and *r*-roscovitine (cyc202) in treatment exhibited even stronger inhibitory activities in HIV-1 infected PBMCs.

## Background

Human immunodeficiency virus type 1 (HIV-1) is the causative agent of Acquired Immunodeficiency Syndrome (AIDS). Current therapies are capable of controlling viral infection but do not represent a definitive cure. The virus has proven to be capable of developing resistance to therapy, evading the immune response, altering cellular immune function and protecting infected cells from apoptosis. HIV-1 is inherently capable of accomplishing these functions with a limited genome that expresses only nine proteins. As such, the HIV-1 must make extensive use of cellular pathways and subvert native molecular processes for its own purposes.

Expression of the HIV-1 proviral genome requires host cell transcription factors as well as the Tat viral transactivator (reviewed in [1-3]). Tat stimulates formation of full-length transcripts from the HIV-1 promoter [4,5] by promoting efficient transcriptional elongation (reviewed in [1,2]). Tat interacts with the bulge of the transactivation response element (TAR) RNA, a hairpin-loop structure at the 5'-end of all nascent viral transcripts [6-9]. Full functional activity of Tat requires host cell cofactors, which interacts with the loop of TAR RNA hairpin (reviewed in [1,2]) as well as other site on the LTR. Tat also associates with a protein kinase that phosphorylates the C-terminal domain (CTD) of RNA Polymerase II (RNA Pol II) called Tat associated kinase (TAK) [10]. The CTD consists of heptapeptide repeats, Tyr^1^-Ser^2^-Pro^3^-Tyr^4^-Ser^5^-Pro^6^-Ser^7^, which are phosphorylated on serine 2 (Ser-2) and serine 5 (Ser-5) during transcription [11,12]. Recently, serine 7 (Ser-7) has been shown to be phosphorylated by cdk7 [13,14]. Previously, it has also been shown that partially purified TAK and the loop-binding factor represent the same protein complex, cdk9/cyclin T1 [15-17]. Tat associates with cdk9 [16,17] through interaction with cyclin T1 which interacts with the TAR RNA loop structure [15]. Tat interacts with human cyclin T1 through a critical cysteine and the presence of a different amino acid in this position in rodent cells renders Tat transactivation inefficient [18,19]. In an *in vitro *transcription system, Tat stimulates additional phosphorylation of the hyperphosphorylated RNA Pol II [20]. In kinase assays, Tat induces phosphorylation of CTD by cdk9, which requires the N-terminal domain (amino acids 1 to 48) and the arginine-rich motif (amino acids 49-57) of Tat [21]. Tat may also induce TFIIH-associated cdk7 to phosphorylate Ser-5 in the pre-initiation complex [22,23]. Subsequently, TFIIH dissociates from the preinitiation complex and this dissociation relieves inhibition of cdk9 autophosphorylation [24], which is required for efficient binding of cdk9/cyclin T1 to TAR RNA [21].

Recently, a growing body of evidence has indicated the role of yet another cyclin/cdk complex, namely cyclin E/cdk2, in Tat activated transcription. Cyclin E/cdk2 is the major cyclin/cdk complex whose maximal activity is observed at the late G1/S boundary. Cyclin E/cdk2 has been shown to be important in the transition of G1/S by regulating the release of Rb sequestered factors, including E2F [25]. Given the importance that the G1/S checkpoint plays in viral replication, it is not surprising that HIV-1 viral proteins, like Tat, have been shown to modulate G1/S activity. From our own studies, we have observed the increased kinase activity of cyclin E/cdk2 complexes in HIV-1 latently infected cells due to the loss of the natural cdk inhibitor p21/waf1 [26]. Cdk inhibitor p21/waf1 is normally induced by p53 upon cellular stress and regulates the G1/S transition by inhibiting the activity of cyclin/cdk complexes. Studies from our lab have shown that HIV-1 latently infected T cells do not induce expression of p21/waf1 after injury to the host cell. For instance, flow cytometric analysis revealed that upon γ-irradiation, these cells proceeded into the S phase and apoptosed. The lack of p21/waf1 expression was attributed to the physical and functional interaction of Tat with p53, resulting in the inactivation of p53 [26,27]. To further validate the significance of the G1/S and cdk2 in HIV-1 transcription *in vivo*, HLM-1 cells (HIV-1^+^/Tat^-^), were first transfected with wild type Tat and were subsequently blocked with either hydroxyurea (a general G1/S blocker) or nocodazole (a general M phase blocker). Supernatants were collected every third day and analyzed for the presence of the gag/p24 antigen. HIV-1 attained peak viral replication between days 9 and 12 for those cells blocked with nocodazole, while G1/S blockage by hydroxyurea resulted in the dramatic inhibition of virion production [28]. Collectively, these studies pointed to two important findings. One, that HIV-1 in latently infected cells down modulates the natural cdk inhibitor p21/waf1 (i.e., by Tat binding to p53 and/or other related mechanisms), and in turn is able to control the primary cdk target such as cyclin E/cdk2 complex, and second, that G1/S kinases, such as cdk2/cyclin E, could be targeted for inhibition of HIV-1 replication using drugs that mimic the natural cdk inhibitors.

Over the past few years, pharmacological cdk inhibitors (PCIs) have been reported to prevent viral replication *in vitro *[29]. The underlying mechanism of action, inhibition of cellular rather than viral targets, is unlikely to favor the appearance of resistant strains and could potentially be efficient against several unrelated viruses. Numerous viruses require active cdks for their replication and some viruses actually encode their own cyclins, thereby regulating their host cell cycle [30]. Cdks are required for replication of viruses that multiply only in dividing cells, such as adeno- and papillomaviruses. Recently, cdks have also been shown to be required for the replication of viruses that multiply in non-dividing cells, such as HIV-1 and herpes simplex virus types 1 and 2 (HSV-1 and -2) [31,32]. In these experiments PCIs were shown to have potent antiviral activity *in vitro *against HIV-1, HSV-1 and -2, human cytomegalovirus, varicella-zoster virus, and to inhibit specific functions of other viruses [33]. Since two PCIs, flavopiridol and roscovitine, have been proven to be non-toxic in human clinical trials against cancer [34], PCIs, therefore may be useful as antivirals. As significant advantage of PCI are its activity against many viruses, including drug-resistant strains of HIV-1 and HSV-1 [35,36]. Furthermore, the antiviral effects of a PCI and a conventional antiviral drug could have an additive effect. Roscovitine is the second-best-studied PCI *in vivo *(after flavopiridol) and it has proven non-toxic in several animal models [37,38]. The purified *r*-enantiomer of roscovitine (cyc202) has entered human clinical trials. In phase I clinical trials, *r*-roscovitine has proven to be orally bioavailable and to have no acute toxicity [39].

Other class of inhibitors including paullones represents a novel class of small molecule cdk inhibitors. Paullones constitute a new family of benzazepinones with promising antitumoral properties. They were described as potent, ATP-competitive, inhibitors of the cell cycle regulating cdks [40]. Alsterpaullone, the most active paullone, was demonstrated to act by competing with ATP for binding to GSK-3β. Alsterpaullone inhibits the phosphorylation of tau *in vivo *at sites which are typically phosphorylated by GSK-3β in Alzheimer's disease [41]. Alsterpaullone also inhibits the cdk5/p35-dependent phosphorylation of DARPP-32 in mouse striatum slices *in vitro *[41]. This dual specificity of paullones may turn these compounds into very useful tools for the study and possibly treatment of neurodegenerative and proliferative disorders [42]. Replacement of the 9-bromo substituent of kenpaullone by a 9-cyano or 9-nitro group produced a substantial increase in enzyme-inhibiting potency [43]. Interestingly, alsterpaullone has been selected for preclinical development in a NCI program [44].

In this study, we identified alsterpaullone having a potent inhibitory effect on HIV-1 infected cells. Its mechanism of action has previously been attributed to inhibition of cdk2/cyclin A complex at the G1/S as well as few other kinases. Here, the primary mode of the inhibition in infected cells appears to be at the protein levels of cyclins which ultimately result in apoptosis of HIV-1 infected cells. Finally, low concentration of two drugs combined, alsterpaullone and *r*-roscovitine, favor inhibition of the HIV-1 transcription in primary cells.

## Results

### Screening of twenty-four inhibitors in HIV-1 infected and uninfected cells

We analyzed the effects of twenty four different cdk inhibitors in HIV-1 infected cells ACH2, OM10.1, J1-1, U1 and uninfected cells including CEM, Jurkat and U937 cells. For the initial set of screenings, cells were cultured in medium (0.5 × 10^6 ^cells/well) with inhibitors at 10 μM concentration. After 72 hours of culture, cell viability was determined using trypan blue exclusion method. Results of such a screen are shown in Table 1 where percent of live cells are indicated after various drug treatments. A total of ~100 cells that were not clumped together were counted and scored with trypan blue. The inhibitors were classified into three categories: high, moderate or poor selectivity according to their cellular viability in both HIV-1 infected and uninfected cells. Among the 24 inhibitors, alsterpaullone proved to be the drug with the highest selectivity in promoting cell death in HIV-1 infected cells, followed by indirubin-3-monoxime, indirubin-3-monoxime-5-indo, purvalanol A, and *r*-roscovitine. Along these lines, we have previously shown that *r*-roscovitine (cyc202) is able to inhibit virus replication both in primary cells as well as in cells lines *in vitro*. Also, there were varying levels of cell death in uninfected treated cells; however drugs in the high selectivity category were generally more active toward HIV-1 infected cells. All infected cells expressed some levels of doubly or singly spliced messages when cultured in 10% fetal calf serum. Collectively, these preliminary cell based screening data indicated that some cdk inhibitors may be more selective toward HIV-1 infected cells and promote cell death *in vitro *as compared to uninfected cells.

**Table 1:** Screening of various cdk inhibitors and related molecules in HIV-1 infected cells

Selectivity	Name		ACH2	J1.1	OM10.1	U1	CEM	Jurkat	U937
			Infected				Uninfected		
**High**	Alsterpaullone	(10 μM)	11	25	15	37	89	92	88
	Indirubin-3'-monoxime	(10 μM)	22	32	35	38	84	83	87
	Indirubin-3'-monoxime-5'-indo	(10 μM)	24	35	37	52	80	82	80
	Purvalanol A	(10 μM)	27	53	48	54	78	79	77
	*r*-Roscovitine	(10 μM)	32	40	30	35	75	85	82

**Moderate**	CGP 74514A	(10 μM)	42	56	54	52	72	77	70
	Aloisine A	(10 μM)	50	52	59	53	72	70	68
	Bohemine	(10 μM)	58	65	67	50	72	80	50
	2,6-Diaminopurine	(10 μM)	64	75	77	74	75	68	65
	2,6-Dichloropurine	(10 μM)	75	74	76	75	69	71	71
	Flavone	(10 μM)	86	85	83	85	75	67	50

**Poor**	6-Benzyloxypurine	(10 μM)	90	91	92	88	68	72	54
	Compound 52	(10 μM)	95	97	94	95	97	97	98
	9-Cyanopaullone	(10 μM)	97	97	95	98	95	96	97
	6-Dimethylaminopurine	(10 μM)	95	95	96	97	97	95	96
	Indirubin-3'-monoxime-5'-sulphonic acid	(10 μM)	95	95	96	96	96	96	95
	Iso-olomoucine	(10 μM)	96	96	96	97	95	95	97
	N-6-(Δ2-Isopentenyl)-adenine	(10 μM)	96	96	98	99	95	98	96
	Kenpaullone	(10 μM)	94	97	95	95	95	95	95
	Olomoucine	(10 μM)	95	95	96	96	95	95	95
	Olomoucine N9-isoppropyl	(10 μM)	96	95	95	96	95	96	96
	*s*-Roscovitine	(10 μM)	95	95	95	94	95	98	96
	WHI-P180	(10 μM)	95	97	98	99	98	97	98
	SC-514	(10 μM)	95	98	99	99	98	96	99

### Alsterpaullone exhibited an inhibition of cell viability and promoter activity in HIV-1 infected cells

**Figure 1:**
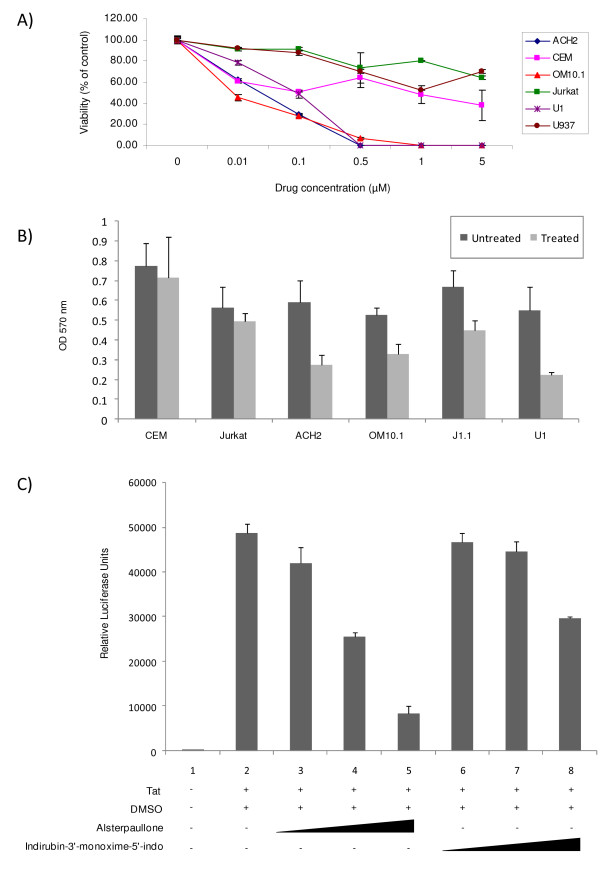
**Infected cell viability and Tat-induced HIV-1 LTR transcription are inhibited by alsterpaullone**. **A) **HIV-1 infected cells (ACH2, OM10.1, and U1) and corresponding control uninfected cells (CEM, Jurkat and U937) were plated in 24-well plates and cultured with increasing concentrations of alsterpaullone (0.01-5 μM). After 48 hours, the cells were stained by trypan blue and percent viability calculated with a hemocytometer. Assays were performed in triplicate, average values and standard deviations are shown. **B) **MTT assays were used for HIV-1 infected and corresponding control uninfected cells. Cells were seeded in a 96-well plate and cultured with 0.25 μM alsterpaullone, and 48 hours later, absorbance was read at 570 nm. Percent viability assays were performed in triplicate and average values and standard deviations are shown. **C) **TZM-bl cells were transfected with 1 μg of Tat and treated the next day with DMSO or the indicated compound (50, 150, or 300 nM). Cells were processed 48 hours post drug treatment for luciferase assays. Assays were performed in triplicate, average values and standard deviations are shown.

Following the identification of alsterpaullone as the drug with the highest selectivity in inhibiting HIV-1 infected cells, we next decided to look at its effect in a dose dependent manner on HIV-1 infected cells at different concentrations including 0.01, 0.1, 0.5, 1.0, and 5 μM. As shown in Figure 1A, after cell treatment with various concentrations for 3 days, the inhibition of cell viability in HIV-1 infected cells was more pronounced when compared to the control uninfected group. We normalized for the percent of live cells for each cell type at time zero and performed triplicates for each concentration. The CC_50 _of alsterpaullone was determined to be at ~0.10-0.25 μM for the HIV-1 infected cells and ~5 μM for the uninfected cells. To further refine and validate the results in panel A, we used an MTT assay in cells treated with a fixed concentration of the drug (0.25 μM). Results in panel B show that by and large, infected cells are more susceptible to alsterpaullone as compared to uninfected cells. Finally we asked whether alsterpaullone was able to inhibit Tat activated transcription in an LTR-reporter assay. TZM-bl cells contain an integrated HIV-1 LTR-luciferase reporter construct and were transfected with Tat and treated with various concentrations of alsterpaullone, and indirubin-3'-monoxime-5'-indo as control. Luciferase assays revealed that alsterpaullone, indirubin-3'-monoxime-5'-indo and purvalanol A (data not shown) decreased viral transcription of the fully chromatinized promoter at an approximate IC_50 _of 150 nM or less (Figure 1C). Collectively, these results imply that alsterpaullone can selectively inhibit HIV-1 promoter activity and kill infected cells in a dose dependent manner.

### Effect of alsterpaullone on cdk2/cyclinA activity in HIV-1 infected and uninfected cells

**Figure 2:**
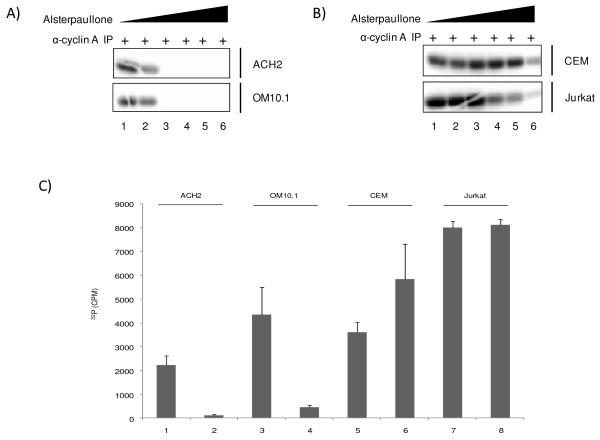
**Alsterpaullone inhibition of cdk2/cyclin A complex in HIV-1 infected cell**. **A) **Equal amount (2 mg) of cytoplasmic proteins from alsterpaullone-treated ACH2 and OM10.1 cells were immunoprecipitated with anti-cyclin A antibody and the cdk2 activity was examined by *in vitro *kinase assay using histone H1 as a substrate. Alsterpaullone at various concentrations (0.01, 0.1, 0.5, 1, 5, and 25 μM) were used in treatment of cells. The [γ-^32^P]-labeled histone H1 was visualized by autoradiography. Alsterpaullone completely inhibits cdk2 kinase activity in infected cells at 0.5 μM (lane 2). **B) **Similar to panel A, but used extracts from uninfected cells for IP. Alsterpaullone moderately inhibited cdk2 activity in uninfected CEM and Jurkat cells but only at high concentrations (lanes 4-6). **C) **Effect of low concentration of alsterpaullone in kinase assay. Similar to panel A and B, a low concentration of alsterpaullone (0.5 μM) was used in kinase inhibition studies. Infected (ACH2 and OM10.1) as well as uninfected control (CEM and Jurkat) cell lysates were used for these assays. Lanes 1, 3, 5 and 7 were cells treated with DMSO and lanes 2, 4, 6, and 8 were treated with alsterpaullone. Results are triplicate experiment of using cyclin A IP as the Kinase and histone H1 as the substrate.

Alsterpaullone was previously tested on a variety of highly purified kinases *in vitro *[41]. Kinase activities were assayed with appropriate substrates, cold ATP (15 μM) as control, and in the presence of increasing concentrations of alsterpaullone. The IC_50 _values were obtained from the dose-response curves. Most kinases tested were poorly or not inhibited (IC_50 _> 10 μM). However, in addition to the previously reported effect on cdk1/cyclin B, alsterpaullone was found to inhibit cdk2/cyclin A, cdk2/cyclin E, cdk5/p35 and GSK-3α/GSK-3β (IC_50 _values of 15, 200, 40 and 4 nM respectively). We therefore asked which of these various cdk/cyclin complexes in HIV-1-infected cells were most sensitive to alsterpaullone. A typical kinase assay from HIV-1 infected (OM10.1 cells) and uninfected cells (Jurkat and CEM cells) is shown in Figure 2. Alsterpaullone (0.01, 0.1, 0.5, 1, 5, and 25 μM) treated cells were immunoprecipitated with cyclin A antibody, isolated complexes were washed and added to kinase reactions containing histone H1 as a substrate. As seen in Figure 2A, 0.5 μM of alsterpaullone completely inhibited the cdk2 kinase activity from infected cells when using histone H1 as a substrate (Figure 2A, lane 3). The cdk2 activity however was inhibited at much higher alsterpaullone concentrations in uninfected cells (Figure 2B, lanes 4-6). As a negative control, kinase assays were performed with immunoprecipitation with anti-IgG antibody with minimal background activity (data not shown). To further validate these results, we performed kinase assays with fixed concentration of alsterpaullone (0.5 μM) and found a reproducible pattern where kinase activity was severely inhibited in immunoprecipitates from infected and not the uninfected cells (Figure 2C). Collectively, these data indicates that cdk2 in HIV-1 infected cells may be either more sensitive to alsterpaullone or the expression levels in these cells may have changed following drug treatment.

### Inhibitory effect of alsterpaullone on cyclin/cdk expression

**Figure 3:**
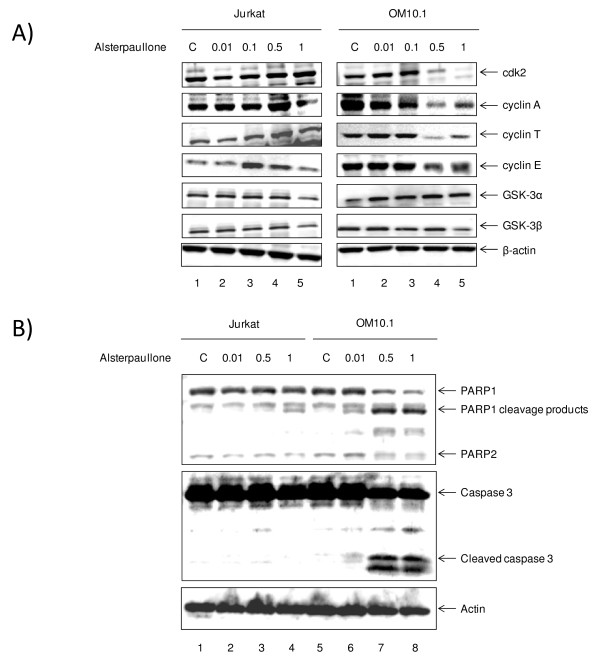
**Alsterpaullone inhibition of cdk2 and cyclin expression in HIV-1 infected and uninfected cells**. **A) **HIV-1 infected OM10.1 and uninfected Jurkat cells were treated with alsterpaullone (0.01, 0.1, 0.5, and 1 μM) for 48 hours. Total cell extracts (25 μg) were subjected to western blot analysis for cdk2, cyclin A, cyclin E, cyclin T, GSK-3α, GSK-3β, and actin. **B) **Similar to panel A, Jurkat and OM10.1 cells were treated with various concentrations of alsterpaullone and cell extracts were processed for presence of apoptosis markers (cleaved PARP and caspase-3 products). β-actin was used as internal control for all westerns. Both processed and cleaved PARP and caspase-3 were observed in higher concentrations of alsterpaullone treated OM10.1 infected cells.

Because alsterpaullone is a purine analog, it can compete with the ATP binding site in cdks and has been shown to inhibit cdk2/cyclin E and cdk2/cyclin A kinase activities with an IC_50 _at 0.035 and 0.07 μM, respectively when using *in vitro *kinase assays. To examine whether alsterpaullone inhibits expression of these cell cycle regulatory proteins in HIV-1 infected cells, we determined the levels of cdk2, cyclin E, cyclin A, and other kinases by western blot analysis. As shown in Figure 3A, the levels of cdk2, and cyclin A expression declined dramatically at 0.5 μM of alsterpaullone treatment in infected OM10.1 cells (Figure 3, lane 4). The level of cyclin T and E expression also declined to lower levels in these cells. Therefore, in relations to the previous IP/kinase assays (Figure 2), these results indicate that alsterpaullone down-regulates the amount of functional cdk2/cyclin A complex by reducing the expression/protein levels in HIV-1 infected as compared to uninfected cells.

Next, to determine the efficacy of alsterpaullone in induction of apoptosis in infected cells, we analyzed two markers of apoptosis, namely the cleavage of caspase-3 and PARP using western blot analysis. Both infected and uninfected cells were treated with various concentration of the drug and whole cell extracts were processed for presence of cleaved products. As shown in Figure 3B, the levels of both cleaved PARP and caspase-3 increased in infected cells at 0.5 and 1 μM concentrations. Importantly, alsterpaullone treatment did not significantly induce cleavage of caspase-3 and PARP in uninfected Jurkat cells. Collectively these results indicate that treatment of HIV-1 infected cells with low concentrations of alsterpaullone may result in increase of apoptosis markers in infected cells with little to no apparent apoptosis in uninfected cells.

### Effect of alsterpaullone on the cell cycle and apoptosis in infected and uninfected cells

**Figure 4:**
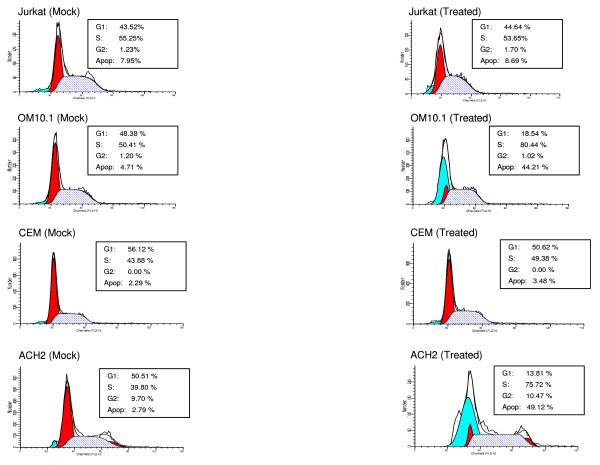
**Alsterpaullone induces apoptosis in HIV-1 infected cells**. For fluorescence-activated cell sorting (FACS), both untreated and treated Jurkat, OM10.1, CEM and ACH2 cells were stained with a mixture of propidium iodide buffer followed by cell sorting analysis. The left panels show mock-treated cells (DMSO) and the right panels correspond to alsterpaullone treated cells (0.5 μM) for 48 hours. A higher percentage of apoptotic cells were observed in treated OM10.1 (~44%) and ACH2 (~48%) cells as compared to untreated, uninfected Jurkat (~7%) and CEM (~3%) counterparts.

We next were interested in determining whether the cell cycle stage of infected cells could be altered after drug treatment. For this we treated both uninfected (Jurkat and CEM) as well as infected (OM10.1 and ACH2) cells with alsterpaullone (0.5 μM) for 48 hours followed by FACS analysis using propidium iodide staining. We had initially performed a pilot experiment with time and drug titrations to find a window of time where cells would begin the process of apoptosis, but no completely progress into final stages of apoptosis (data not shown). Results in Figure 4 show that Jurkat or CEM uninfected cells were not dramatically altered in their cell cycle stages before or after treatment. However, both OM10.1 and ACH2 infected cells were altered in their G1, S, and sub G1 (apoptosis) peaks following drug treatment. Both infected cell types displayed an increase in their G1 population, an increase in S phase, as well as a dramatic increase in apoptotic peaks. No viral particles as assayed by presence of RT were observed in the supernatant after drug treatment (data not shown). These results imply that the apoptotic peaks (more than 10 fold in each infected cell type) could be either coming from the G1 population or partly from the S phase population (loss of G1/S check point). The apparent loss of check point control may be from inactive p53 function and a decrease in p21/waf1 levels in both infected cell types [45-47]. Collectively, these results indicate that the drug effect is mostly specific to G1 and S phase population in HIV-1 infected cells.

### Effect of alsterpaullone in PBMC infected cells

**Figure 5:**
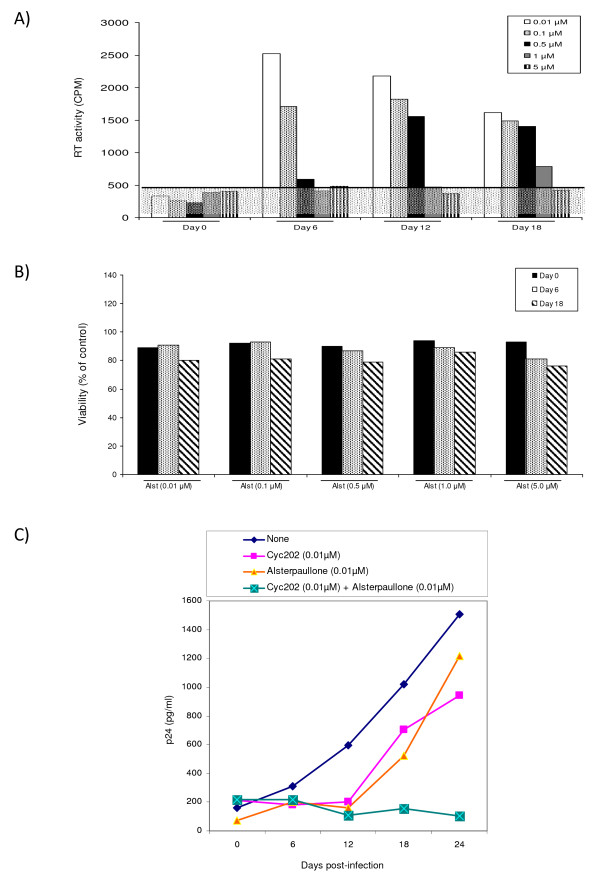
**Effect of alsterpaullone in PBMC infection**. **A) **Phytohemagglutinin (PHA) and IL-2 activated PBMCs were kept in culture for 2 days prior to infection. Approximately 5 × 10^6 ^PBMCs were infected with pNL4-3 (MOI:1). Alsterpaullone treatment (0.01-5.0 μM) was used (only once) immediately after the addition of the fresh medium. Samples were collected every sixth day and stored at -20°C for further analysis (RT assay). Both PHA and IL-2 were added to media every 3 days. Viral supernatants (10 μl) were incubated in a 96-well plate with reverse transcriptase (RT) reaction mixture, incubated overnight at 37°C, spotted, washed, dried, and then counted using a Betaplate counter. **B) **Cells were also counted (~100/date) for viability using trypan blue staining method. **C) **Approximately 5 × 10^6 ^activated PBMCs were infected with primary HIV-1 strain (THA/92/00NSI), and then treated after viral adsorption (12 hrs) with either mock (DMSO) or cyc202 (0.01 μM) or alsterpaullone (0.01 μM) or with the combination of both drugs. Samples were collected every six days (0, 6, 12, 18 and 24 days) and stored at -20°C or p24 assay.

We next performed an infection of PHA and IL-2 activated PBMCs and treated these cells with various concentrations of alsterpaullone for up to 18 days. In this primary cell system, both the effect of HIV-1 replication (using RT assay) and the percent of live cells (trypan blue exclusion) were used to monitor the infection. As seen in Figure 5A, 1 μM of alsterpaullone almost completely inhibited virus replication at day 12 and inhibited replication by approximately 50% at day 18 in two independent experiments. It is important to note that drug treatment was performed only once in these cells (addition at day 0). Furthermore, concentrations up to 5 μM did not alter the percent of live cells in either uninfected or infected cell types (panel B) indicating that low concentrations of the drugs are not toxic to primary activated cells.

Next, we asked whether low concentrations of *r*-roscovitine and alsterpaullone could potentially inhibit virus replication in primary cells. We have previously shown that *r*-roscovitine (cyc202) is able to inhibit virus replication both in primary cells as well as cells lines [48]. The IC_50 _in latent infected cells was from 0.36 μM to 1.8 μM depending on the cell type. Here we utilized a combination of a low 0.01 μM concentration of each *r*-roscovitine and alsterpaullone, which normally would not inhibit viral replication when used in monotherapy. Results in Figure 5C indicate that the addition of low concentrations of both drugs effectively inhibited a field isolate of HIV-1 in PBMC infections. The combination of these two drugs at such low concentrations had no apparent toxic effects in active PBMCs (data not shown). Collectively, these results imply that cdk2 inhibitors that target the G1/S (cyc202) and early S (alsterpaullone) phases may effectively block viral replication in primary cells when infected with HIV-1 field isolates.

## Discussion

In contrast with the latest progress in the understanding of HIV-1 infection, its pathogenesis and mechanism of action-especially in relation to therapies, are still at its infancy. However few well established pathways including cell signaling involving kinases and markers of cell cycle progression have been shown to be tightly regulated in HIV-1 infected cells and therefore provide viable targets for treatment. Cdks are attractive targets for drug development since their activity, required for the correct timing and ordering of the cell cycle, is frequently deregulated in cancer. Numerous small molecule inhibitors of cdks have been identified and proven effective in treating tumors. This is mainly due to the increased sensitivity of the transformed cells to inhibitors and to the changes that are associated with cdk activity and levels in a cell. However the consequences of cdk inactivation are complex and can result in disparate outcomes depending on the tumor type and the genetic context that drives their expression.

We investigated whether targeting the cdk/cyclin axis could inhibit the growth of HIV-1 infected cells and assessed this hypothesis using multiple cdk inhibitors. Along these lines, we searched for various inhibitors targeting multiple cdk/cyclin pathways using published literature and our own search by means of small libraries of compounds. We selected first generation inhibitors with low-high IC_50 _in various cell types and identified their cell growth inhibition efficiencies in HIV-1 infected and uninfected cells. Results in Table 1 clearly show that there are various compounds that specifically target HIV-1 infected cells. In the high selectivity group, alsterpaullone demonstrated the best selectivity to block viability of all HIV-1 infected cells tested and little blockage to control cells at the concentrations tested. Indirubin-3'-monoxime, indirubin-3'-monoxime-5-indo, purvalanol A and *r*-roscovitine also inhibited growth of infected cells to varying degree. Consequently, we decided to focus and study the mechanism of alsterpaullone in the current manuscript.

Our results with titration of alsterpaullone showed that HIV-1 infected cells were more vulnerable to apoptosis in a concentration dependent manner. Many of these so called latent infected cells harbor various forms of virus and have a certain level of leakiness and expression of singly and doubly spliced messages in the absence of any inducers. Therefore, there is viral transcription in many of these cells especially when they are treated and fed with 10% fetal bovine serum, which provides enough cytokine and growth factor signaling to produce leaky viral transcription in these cells.

We then focused on the cdk2/cyclin A complex since it has been shown to be involved in early S phase transition of cell cycle, is important for cellular DNA synthesis, and is a target of alsterpaullone. Interestingly, when we used immunoprecipitation to detect the kinase activity of endogenous cdk2/cyclin A, we found great inhibition with alsterpaullone in infected cells. However, upon western blot analysis of cdk2 and other cyclins in drug treated cells, we found lower levels of cdk2 and cyclins in infected cells and not in uninfected cells. Downregulation of cdk2, cyclin A, cyclin T, and cyclin E in infected cells is interesting and may indicate that cdk/cyclin complexes in HIV-1 infected cells are inherently different in their behavior, partner binding or post-translational modifications, among other factors, which may contribute to its high sensitivity to alsterpaullone. Consistent with the cleaved caspase-3 and PARP levels, FACS analysis also showed a dramatic difference in infected versus uninfected cells. Results in Figure 4 clearly show that, in infected cells (OM10.1 and ACH2), the G1 phase population has decreased and the S phase population has increased, as well as an increase of almost ten-fold in the apoptotic population. This implies that the G1/S checkpoint in infected cells is either non-existent or severely defective which may be the ultimate mechanism of how these cdk inhibitors kill HIV-1 infected cells. Importantly, there was no viral release after treatment of the infected cells with alsterpaullone (data not shown) even though the cells were apoptosing.

When using primary cells, we found similar IC_50 _of inhibition in infected PBMCs as well as an additive effect of *r*-roscovitine (cyc202) with low concentrations of alsterpaullone. Both of these drugs, which target G1/S and the early S phase at low concentrations, do not kill infected or uninfected cells. However, the addition of low concentrations of both drugs to the infected cells selectively inhibits viral replication in primary cells. We therefore concluded that to inhibit HIV-1 activated transcription, one may need to use multiple cdk inhibitors that inhibit critical cdk/cyclin complexes that are needed for HIV-1 transcription, and low concentrations of these drugs may have a synergistic effect in infected cells.

Finally, alsterpaullone is also a potent GSK-3α/GSK-3β inhibitor [49]. GSK-3α/GSK-3β are implicated in the regulation of glycogen synthesis, the Wnt signaling pathway, cell cycle control, transcriptional regulation, and apoptosis [50]. The ability GSK-3α/GSK-3β to regulate this vast array of cellular processes may be related to its numerous substrates including, glycogen synthase, axin, β-catenin, APC, cyclin D1, c-Jun, c-myc, C/EBPα/β, NFATc, RelA and CREB to name a few [50,51]. Interestingly, Tat induces GSK-3β activity, which can be reversed by the addition of the GSK-3β inhibitor lithium [52]. Furthermore, the GSK-3β inhibitors lithium and VPA can protect against Tat and gp120 mediated neurotoxicity [53-55]. Sui *et al. *investigated the role of GSK-3β in NF-kB regulated neuronal apoptosis [56]. They found that neurons exposed to HIV_ADA_-macrophage conditioned medium (MCM) displayed decreased NF-kB activity in a Tat dependent manner. GSK-3β inhibition through the lithium or indirubin treatment blocked NF-kB inhibition, the suppressive binding of RelA to HDAC3, and neuronal apoptosis [56]. Lithium treatment also inhibits HIV-1 replication of both T- and M-tropic viruses in PBMCs as well as TNF stimulated J1.1 cells [57]. Therefore, the inhibition of GSK-3β may have implications for the treatment of neuroAIDS as well as in the inhibition of HIV-1 replication in PBMCs. Future experiments will shed light on the mechanism of inhibition in various viral strains and its possible tropism in infected cells.

## Conclusion

PCIs may be ideal candidates for HIV-1 transcription inhibition, since they target non-essential cellular proteins and avoid emergence of mutant resistant viruses. We previously reported that *r*-roscovitine (a first generation PCI) is a potential inhibitor of HIV-1 replication. PCIs are among the most promising novel antiviral agents to emerge over the past few years. In the current work, we evaluated twenty four cdk inhibitors for their effect on HIV-1 replication *in vitro *and found that alsterpaullone is a potent inhibitor of HIV-1 transcription. FACS analysis showed a more dramatic difference in apoptosis of infected versus uninfected cells, where the G1 phase population has decreased and the S phase population has increased. This implies that the G1/S checkpoint in infected latent cells is either non-existent or severely defective which may be the ultimate mechanism of how these cdk inhibitors kill HIV-1 infected cells.

## Methods

### Cell lines and reagents

The latently HIV-1-infected promyelocytic OM10.1 cell line, the latently infected promonocytic U1 cell line and the uninfected corresponding HL-60 and U937 cell lineages, as well as infected J1-1, ACH2 and their uninfected counterparts Jurkat and CEM (12D7) cells were cultured at 37°C up to 1 × 10^5 ^cells per ml (early log phase of growth) in RPMI-1640 medium supplemented with heat-inactivated fetal bovine serum (10%), streptomycin, penicillin antibiotics (1%) and L-glutamine (1%) (Gibco/BRL, Gaithersburg, MD, USA). OM10.1, ACH2, J1-1 contain a single integrated copy of HIV-1 genome, whereas U1 cells harbor two copies (one wild type and one mutant) of the viral genome in parental U973 cells.

### Cdk inhibitors

The cdk inhibitors used in this study were: aloisine A (270-385-M001), alsterpaullone (270-275-M001), bohemine (270-390-M001), CGP74514A (270-391-M001), compound 52 (270-248-M001), 9-cyanopaullone (270-282-M001), 6-dimethylaminopurine (480-050-M100), indirubin-3'-monoxime (270-271-M001), 5-iodo-indirubin-3'-monoxime (270-424-M001), N-6-(Δ2-Isopentenyl)-adenine (350-034-M100), kenpaullone (270-274-M001), olomoucine (350-013-M005), N9-isopropylolomoucine (270-397-M001), purvalanol A (270-246-M001), (*r*)-roscovitine (350-251-M001), (*s*)-roscovitine (350-293-M001) were purchased from Alexis Co. (San Diego, CA, USA). 6-benzyloxypurine (387606), 2,6-diaminopurine (247847), 2,6-dichloropurine (D73103), flavone (F2003) were purchase from Sigma-Aldrich (St. Louis, MO, USA). Indirubin-3'-monoxime-5-sulfonic acid (402088), iso-olomoucine (495622), WHI-P180 (681500) were purchased from Calbiochem (La Jolla, CA, USA). The cdk inhibitor, flavopiridol was a gift from Dr. Ajit Kumar at The George Washington University Medical Center. All inhibitors were prepared in 10 mM stock solution. 2,6-dichloropurine and diethylmaleate were dissolved in ethanol, flavone was dissolved in acetone, flavopiridol and pyrrolidinedithiocarbamic acid were dissolved in water and 5-aminosalicylic acid was dissolved in hydrochloric acid. All other inhibitors were all dissolved in DMSO.

### Drug screening and cell counting

The initial screening assays included use of HIV-1 infected and uninfected cells that were treated with 24 inhibitors at four concentrations including 0.01, 0.1, 0.5, 1, 5, and 10 μM. Two to six days after treatment (experiment-dependent), cell viability was primarily determined by trypan blue exclusion as well as change of color in media from both infected and uninfected cells. Cells (~100 cells that did not clump) were counted for the number of non-viable cells every 24-48 hours. Subsequent focusing experiments used MTT and flow data to check for viability and apoptosis.

### Protein extracts and immunoblotting

Nuclear and cytoplasmic extracts from uninfected and infected cells were prepared. Cells were collected, washed once with PBS and pelleted. Cells were lysed in a buffer containing containing Tris-HCl pH 7.5, 120 mM NaCl, 5 mM EDTA, 0.5% NP-40, 50 mM NaF, 0.2 mM Na_3_VO_4_, 1 mM DTT and one tablet complete protease inhibitor cocktail per 50 ml. Lysis was performed under ice-cold conditions, incubated on ice for 30 minutes and spun at 4°C for 5 minutes at 14,000 rpm. The protein concentration for each preparation was determined with a Bio-Rad protein assay kit (Bio-Rad Laboratories, Hercules, CA, USA). Cell extracts were resolved by SDS PAGE on a 4-20% tris-glycine gel (Invitrogen, Carlsbad, CA, USA). Proteins were transferred to polyvinylidene difluoride microporous membranes using the iBlot dry blotting system as described by the manufacturer (Invitrogen). Membranes were blocked with Dulbecco's phosphate-buffered saline (PBS) 0.1% Tween-20 + 3% BSA. Primary antibody against specified proteins was incubated with the membrane in blocking solution overnight at 4°C. Antibodies against cdk2 (M-2), cyclin E (M-20), cyclin A (H-432), poly (ADP-ribose) polymerase PARP 1/2 (H-250), caspase-3 (H-277), and actin (C-11) were purchased from Santa Cruz Biotechnology (Santa Cruz, CA, USA). Cyclin T1 (ab27963), GSK3-α (9338), and GSK3-β (9332) antibodies were obtained from Cell Signaling Technology, Inc. (Danvers, MA, USA). Membranes were washed twice with PBS + 0.1% Tween-20 and incubated with HRP-conjugated secondary antibody for one hour in blocking solution. Presence of secondary antibody was detected by SuperSignal West Dura Extended Duration Substrate (Pierce, Rockford, IL, USA). Luminescence was visualized on a Kodak 1D image station (Carestream Helath, Rochester, NY, USA).

### Immunoprecipitation and in vitro kinase assay

For immunoprecipitation (IP) 2 mg of extract from alsterpaullone-treated (0-5.0 μM) CEM, ACH2, OM10.1 and Jurkat cells were immunoprecipitated at 4°C overnight with cyclin A antibody. The next day complexes were precipitated with A/G beads (Calbiochem) for two hours at 4°C. IPs were washed twice with appropriate TNE buffer and kinase buffer. Reaction mixtures (20 μl) contained final concentrations: 40 mM β-glycerophosphate pH 7.4, 7.5 mM MgCl_2_, 7.5 mM EGTA, 5% glycerol, [γ-^32^P]ATP (0.2 mM, 1 μCi), 50 mM NaF, 1 mM orthovanadate, and 0.1% (v/v) β-mercaptoethanol. Phosphorylation reactions were performed with IP material and 200 ng of histone H1 in TTK kinase buffer containing 50 mM HEPES (pH 7.9), 10 mM MgCl_2_, 6 mM EGTA, and 2.5 mM dithiothreitol. Reactions were incubated at 37°C for 1 hour and stopped by the addition of 1 volume of Laemmli sample buffer containing 5% β-mercaptoethanol and ran on a 4-20% SDS-PAGE. Gels were subjected to autoradiography and quantitation using a Molecular Dynamics PhosphorImager software (Amersham Biosciences, Piscataway, NJ, USA).

### MTT Viability Assay

Five thousand cells were plated per well in a 96-well plate and the next day cells were treated with 0.25 μM alsterpaullone or DMSO. Forty-eight hours later, 10 μl MTT reagent (50 mg/ml) was added to each well and plates incubated at 37°C for 2 hours. Next, 100 μl of DMSO was added to each well and the plate was shaken for 15 minutes at room temperature. The assay was read at 570 nM using a SpectraMax 340 plate reader (Molecular Devices, Sunnyvale, CA, USA).

### Flow Cytometry

For cell cycle analysis, cells treated with or without drugs and subsequently collected by low speed centrifugation washed with PBS without Ca^2+ ^and Mg^2+ ^and then fixed with 70% ethanol. For fluorescence-activated cell sorting (FACS) analysis, cells were stained with a mixture of propidium iodide buffer (PBS with Ca^2+ ^and Mg^2+^, 10 μg/ml RNase A, 0.1% Nonidet P-40, and 50 μg/ml propidium iodide) followed by FACS analysis. Cells were washed twice with cold PBS without Ca^2+ ^and Mg^2+^, resuspended in 1 × binding buffer (10 mM HEPES-NaOH pH 7.4, 140 mM NaCl, 2.5 mM CaCl_2_) and 5 μl of propidium iodide/10^5 ^cells, and incubated at room temperature for 15 minutes. Cell histograms were acquired using CELLQuest software (BD Biosciences, Bedford, MA, USA) and analyzed by ModFit LT software (Verity Software House, Topsham, ME, USA). Detection of apoptosis through annexin V and PI staining was done according to the manufacturer's protocol (BD Pharmingen, San Jose, CA, USA). In brief, cells were washed three times in PBS and re-suspended in binding buffer at 1 × 10^6 ^cells/ml. An aliquot of 1 × 10^5 ^ells was stained with annexin V-FITC and PI for 15 minutes at room temperature. Analysis was performed on a BD FacsCalibur flow cytometer. Cells were considered to be early apoptotic if they exhibited staining for annexin V, but not PI. The double positive population was considered to be in the late stage of apoptosis.

### PBMC Infection

Phytohemagglutinin-activated PBMCs were kept in culture with IL-2 for 2 days prior to each infection. Isolation and treatment of PBMCs were performed by following the guidelines of the Centers for Disease Control. Approximately 5 × 10^6 ^PBMCs were infected with either pNL4-3 (MOI:1) or primary HIV-1 strain (THA/92/00NSI; 5 ng of p24 gag antigen). Other HIV-1 mutant viruses (AZT, 3TC, TIBO and protease) were also used for PBMC infections (data not shown). All viral isolates were obtained from the National Institutes of Health AIDS Research and Reference Reagent Program. After 8 hours of infection, cells were washed and fresh medium was added. Drug treatment was performed (only once) immediately after the addition of fresh medium, supernatants from the infected PBMCs were collected and used directly for reverse transcriptase (RT) assays or p24 assays.

### Luciferase Assay

TZM-bl cells were transfected with pc-Tat (1 μg) using the Lipofectamine reagent (Invitrogen) according to the manufacturer's instructions. TZM-bl cells contain an integrated copy of the firefly luciferase gene under the control of the HIV-1 promoter (obtained through the NIH AIDS Research and Reference Reagent Program). The next day, cells were treated with DMSO or the indicated compound at increasing concentrations. Forty-eight hours post drug treatment, luciferase activity of the firefly luciferase was measured with the BrightGlo Luciferase Assay (Promega, Madison, WI, USA) and luminescence was read from a 96 well plate on an EG&G Berthold luminometer (Berthold Technologies, Oak Ridge, TN, USA).

### RT and p24 assays

For RT assays, viral supernatants (10 μl) were incubated in a 96-well plate with RT reaction mixture containing 1× RT buffer (50 mM Tris-HCl, 1 mM DTT, 5 mM MgCl_2_, 20 mM KCl), 0.1% Triton, poly(A) (10^-2 ^U), poly(dT) (10^-2 ^U) and [^3^H]TTP. The mixture was incubated overnight at 37°C and 5 μl of the reaction mix was spotted on a DEAE Filter mat paper (PerkinElmer, Shelton, CT, USA) washed four times with 5% Na_2_HPO_4 _and three times with water, and then dried completely. RT activity was measured in a Betaplate counter (Wallac, Gaithersburg, MD). For p24 assays, supernatants from infected cells were centrifuged for 8 minutes at 1200 rpm to remove contaminating cells. p24 levels in the supernatants were then assayed by enzyme-linked immunosorbent assay (AIDS Vaccine Program, NCI-Frederick Cancer Research and Development Center, Frederick, MD, USA) by following the manufacturer's instructions.

## Competing interests

The authors declare that they have no competing interests.

## Authors' contributions

^†^Both IG and EA share first authorship of the current manuscript. IG performed western blot analysis, transfections, MTT assays and luciferase assays. EA performed drug treatment studies, western blot analysis, cell cycle analysis, kinase assays and virus infections. KK aided in the preparation of the manuscript and in the experimental design. FK coordinated the research, experimental design and drafting of the manuscript. All authors have read and approved the final manuscript.
